# Enhancing the Toxicity of Cypermethrin and Spinosad against *Spodoptera littoralis* (Lepidoptera: Noctuidae) by Inhibition of Detoxification Enzymes

**DOI:** 10.3390/toxics11030215

**Published:** 2023-02-24

**Authors:** Marwa H. El-Sayed, Mohamed M. A. Ibrahim, Ahmed E. A. Elsobki, Ahmed A. A. Aioub

**Affiliations:** 1 Plant Protection Department, Faculty of Agriculture, Zagazig University, Zagazig 44511, Egypt; 2 Plant Protection Institute, Agriculture Research Center, Giza 12619, Egypt

**Keywords:** cypermethrin, spinosad, enzyme inhibitors, detoxification enzymes, *Spodoptera littoralis*

## Abstract

The extensive use of wide-ranging insecticides in agricultural activities may develop resistance in insects. The dipping technique was utilized for examining changes in detoxifying enzyme levels in *Spodoptera littoralis* L. induced by cypermethrin (CYP) and spinosad (SPD) with and without a combination of three enzyme inhibitors: triphenyl phosphate (TPP), diethyl maleate (DEM), and piperonyl butoxide (PBO), at 70 μg/mL. PBO, DEM, and TPP showed 50% mortality against larvae at 236.2, 324.5, and 245.8 μg/mL, respectively. The LC_50_ value of CYP on *S. littoralis* larvae reduced from 2.86 μg/mL to 1.58, 2.26, and 1.96 μg/mL, while the LC_50_ value of SPD declined from 3.27 μg/mL to 2.34, 2.56, and 2.53, with the addition of PBO, DEM, and TPP, respectively, 24 h after treatment. Moreover, the activity of carboxylesterase (CarE), glutathione S-transferase (GST), and cytochrome P450 monooxygenase (Cyp 450) was significantly inhibited (*p* < 0.05) by TPP, DEM, PBO plus CYP, and SPD in *S. littoralis* larvae in comparison with tested insecticides alone. These findings suggested that three enzyme inhibitors play a major role in increasing the toxicity of CYP and SPD in *S. littoralis* and will provide insight into how to overcome insecticide resistance in insects.

## 1. Introduction

The cotton leafworm, *Spodoptera littoralis* (Lepidoptera: Noctuidae), is a common cotton pest that attacks different field crops, causing great losses in yield [1]. It feeds on the leaves of several old- and new-world plant species throughout the year [2]. The overuse of synthetic pesticides against *S. littoralis* has resulted in increasing synthetic insecticide resistance, which has become a crucial issue for successful pest management [3,4,5,6]. Moreover, several environmental and health problems and harmful effects on natural enemies were observed [7,8].

Cypermethrin (CYP) is a synthetic pyrethroid that is widely utilized as an insecticide in crop protection in order to minimize yield loss and maximize yield quality in numerous countries [9]. CYP is a modified derivative of pyrethrins, which are natural substances extracted from the flowers of *Chrysanthemum cinerariaefolium* [10] and are known to affect the nervous system, specifically by deleting the closure of sodium channels, causing repetitive after-discharges that result in hyperexcitation of the nervous system [11]. CYP has low mammalian toxicity [12] and highly effective insecticidal properties against insects from several orders, including Coleoptera, Diptera, Hemiptera, and Lepidoptera [13].

Spinosad (SPD) is a bio-insecticide derived from the fermentation of the bacterium *Saccharopolyspora spinosa* [14]. This insecticide is highly toxic through contact and ingestion of a number of pests in the Lepidoptera order [15]. It affects nicotinic acetylcholine receptors (nAChRs) and gamma amino butyric acid (GABA) receptor sites in the insect nervous system [16]. Unfortunately, the continuous application of CYP and SPD has developed resistance in *S. littoralis*, resulting in the widespread failure of insect control.

Insecticide resistance has been a serious impediment to effective chemical control through the use of traditional insecticides [17,18]. It is well-known that several mechanisms of resistance, including biochemical, genetic, metabolic, and even numerous systems, are involved in insecticide resistance [19]. Insecticide resistance is caused by either an increase in detoxifying enzyme levels or a decrease in target-site sensitivity [20,21]. Insect detoxification can be divided into two stages: phase I and phase II (involving metabolizing enzymes) [22]. The main enzymes included in both phases of detoxification systems are carboxylesterase (CarE), cytochrome P450 monooxygenase (Cyp 450), and glutathione S-transferase (GST) [23,24]. The function of detoxifying enzymes is to protect insects against the toxic effects of pesticides by degrading the insecticides and reducing their action [25]. The importance of Cyp 450-mediated metabolism in resistance to CYP against *S. littoralis* is highlighted [26]. In addition, SPD may have a potential role in controlling *S. littoralis*. Therefore, it is considered a promising tool in integrated pest management programs for controlling cotton leafworm under laboratory conditions [27]. Rotation of SPD with none and negative cross-resistance can effectively control *S. littoralis* [4]. Moreover, the inhibition of detoxification enzymes, representing defensive reactions in insects, plays a key role in reducing resistance in *S. littoralis* [28]. Furthermore, the inhibition of enzymes could be the result of the synergists of the essential oils when mixed with CYP to control *S. littoralis* [1]. Thus, one of the fundamental processes for reducing insect resistance to insecticides is the inhibition of detoxification enzymes [3].

Enzyme inhibitors are substances interacting with enzymes, either temporarily or permanently, in order to prohibit enzymes from functioning correctly [29]. Insecticide synergists significantly contribute to addressing resistance issues in insecticide applications [30]. Three synergists, including triphenyl phosphate (TPP), diethyl maleate (DEM), and piperonyl butoxide (PBO), are routinely utilized as inhibitors of carboxylesterase, glutathione S-transferases, and cytochrome P450 monooxygenase, respectively [31]. Synergists are often utilized in combination with a wide range of pesticide classes in order to enhance control efficacy while reducing treatment rates [32,33]. Thus, several synergistic investigations have revealed that enhanced enzymatic activity is one of the fundamental processes in insect resistance that might block or hydrolyze the pesticide [34,35].

The current study hypothesizes that synergist compounds such as enzyme inhibitors combined with chemical insecticides may be beneficial in overcoming the mechanisms of resistance in insects. Hence, this work aims to assess the toxicity of cypermethrin (CYP) and spinosad (SPD) alone and in combination with PBO, DEM, and TPP as enzyme inhibitors on metabolic enzyme inhibition in the fourth larval instars of *S. littoralis* under laboratory conditions.

## 2. Materials and Methods

### 2.1. Compounds

Cypermethrin (CYP) (20% EC) and spinosad (SPD) (8.4% SC) were obtained from the Central Laboratory of Pesticides (Dokki, Giza, Egypt). Triphenyl phosphate (TPP) (≥99%), diethyl maleate (DEM) (98%), and piperonyl butoxide (PBO) (90%) were purchased from Sigma Aldrich (Germany). CarE, Cyp 450, and GST enzyme kits were obtained from the Egyptian Company for Biotechnology (SAE) (Al-Obour City, Industrial Area, Block 20008, Piece 19A, Cairo, Egypt).

### 2.2. Insect

The *S. littoralis*-sensitive strain was received and maintained from the Laboratory of Insect Population Toxicology Department, Central Agricultural Pesticides Laboratory, Agriculture Research Center (Dokki, Giza, Egypt), under controlled conditions of temperature (25 ± 2 °C), relative humidity (70–80%), and photoperiod (12:12 = L:D). The fourth-instar larvae without exposure to any insecticide, fed with fresh castor bean leaves (*Ricinus communis* L.), were utilized for concentration–mortality assays and biochemical studies.

### 2.3. Bioassays

CYP and SPD at various concentrations against the fourth larval instars of *S. littoralis* were evaluated following the leaf-disc method [36]. Briefly, a series of CYP and SPD concentrations were prepared by making a stock solution, and then serial dilutions were conducted. Leaf discs (0.5 cm × 0.5 cm) were cut from fresh corn leaves and dipped into the test dilution for 10 s, held vertically to allow the excess dilution to drip off, and placed on a rack to dry. Distilled water was utilized as a control. Subsequently, 30 12 h-starved fourth-instar *S. littoralis* larvae were transferred to a 24-well plate. Then, discs were offered to the larvae and left under controlled conditions (25 ± 2 °C) for 24 h. The bioassay was repeated three times. Larvae that could not move when touched with a brush were defined as dead.

### 2.4. Synergism Assays

The effect of synergists (TPP, DEM, and PBO) at 70 μg/mL on the toxicity of CYP and SPD versus *S. littoralis* was determined using the previously mentioned leaf-disc method. Briefly, 12 h-starved fourth-instar larvae were treated with synergists for 1 h. Subsequently, the larvae were shifted to plates and exposed to test compounds at various concentrations. This experiment was conducted three times. Mortality counts were recorded and corrected according to Abbott [37]. The insecticidal activities of tested chemicals were exhibited by the LC_50_ value, as well as the 95% confidence interval [31].

The synergism ratio (SR) was computed according to the following equation reported by Qie et al. [31]: LC_50_ of the larvae treated with insecticide alone/LC_50_ of larvae treated with insecticide plus synergists.

### 2.5. Enzyme Inhibition Assays

#### 2.5.1. Tissue Homogenate Preparation

Based on the bioassay results, living larvae were sampled after the treatment with LC_50_ of CYP and SPD. Individual fourth-instar larvae per treatment were homogenized in 0.1 mol L^−1^ of sodium phosphate buffer (PB) solutions in order to measure the activities of three enzymes as follows: carboxylesterase (CarE, larva was homogenized in 1.0 mL of PB containing 0.1% Triton X-100, pH 7.0), cytochrome P-450 (Cyp 450, 1.0 mL of PB containing 1 mmol L^−1^ of EDTA and 1 mmol L^−1^ of DTT, pH 7.3), and glutathione S-transferases (GSTs, 1.0 mL of PB, pH 7.5). Each enzyme activity was immediately spectrophotometrically assayed.

#### 2.5.2. Carboxylesterase (CarE) Activity

Carboxylesterase (CarE) activity was assayed using a method modified by Van Asperen [38]. Sample aliquots (10 μL) were added to microplate wells with 90 μL of PB (pH 7). Then, a 200 μL mixture of substrate (100 μL of 10 mM α-NA solution) and chromogenic reagent (100 μL of 1 mM Fast Blue RR salt) was added. The reactions proceeded at 37 °C and were measured at 450 nm every 25 s for 10 min.

#### 2.5.3. Glutathione S-Transferases (GSTs) Activity

Glutathione-S-transferase (GST) activity was evaluated by adapting the method as previously described [39]. Here, 1-chloro-2, 4-dinitrobenzene (CDNB) was utilized as a substrate and buffer alone as a blank control. Then, 40 μL of homogenized larvae supernatants in a 520 μL sodium phosphate buffer (0.1 M, pH 7.5) was added with 60 μL of glutathione and 10 μL of CDNB solution. Then, the change in absorbance was immediately recorded for 5 min at 340 nm.

#### 2.5.4. Cytochrome P450 Monooxygenase Activity

Cyp 450 activity was measured using a technique adapted from Hansen and Hodgson [40]. Here, 50 μL of sample was added to microplate wells containing 100 μL of p-NA. Then, 50 μL of 9.6 mmol L^−1^ NADPH was added. For 10 min, the reactions were monitored at 405 nm every 25 s at 27 °C.

### 2.6. Statistical Analysis

LC_50_ values and 95% confidence intervals were calculated by probit analyses using SPSS software (version 19.0, SPSS Inc., Chicago, IL, USA, 2003). GraphPad Prism 5.0 (GraphPad Software Inc., La Jolla, CA, USA) was utilized for plotting the enzymatic activity data, and statistical significance was indicated using one-way ANOVA by comparing all treatments together.

## 3. Results

### 3.1. Insecticidal Activity of Tested Insecticides and Enzyme Inhibitors against the Fourth Larval Instars of S. littoralis

The response of *S. littoralis* larvae exhibited a bioassay result between tested insecticides (CYP and SPD) and enzyme inhibitors (TPP, DEM, and PBO) at 70 μg/mL using the leaf-disc method one day after treatment. As presented in Table 1, Table 2 and Table 3, larvae were more susceptible to CYP and SPD plus enzyme inhibitors (TPP, DEM, and PBO) compared to CYP and SPD alone. The LC_50_ value of CYP was 2.861 μg/mL against the fourth larval instars of *S. littoralis*, whereas the LC_50_ values of CYP plus TPP, DEM, and PBO were 1.962, 2.267, and 1.580 μg/mL, with synergistic ratios of 1.458, 1.262, and 1.810, respectively. When larvae were treated with SPD plus TPP, DEM, and PBO, the LC_50_ value of SPD decreased from 3.273 μg/mL to 2.530, 2.653, and 2.344 μg/mL, with synergistic ratios of 1.293, 1.233, and 1.396, respectively. Based on the above results, all tested inhibitors had a synergistic effect toward the tested insecticides against the *S. littoralis* larvae (Table 3). No significant difference has observed between tested synergists. All three synergists without the tested insecticides exhibited less insecticidal activity to *S. littoralis* larvae, as demonstrated in Table 2. The LC_50_ values of TPP, DEM, and PBO were 245.8, 324.5, and 236.2 μg/mL against the fourth larval instars of *S. littoralis*.

### 3.2. Effects of Tested Insecticides and Enzyme Inhibitors on Carboxylesterases, Glutathione S-Transferases, and Cytochrome P450 Monooxygenase Activity

**Figure 1:**
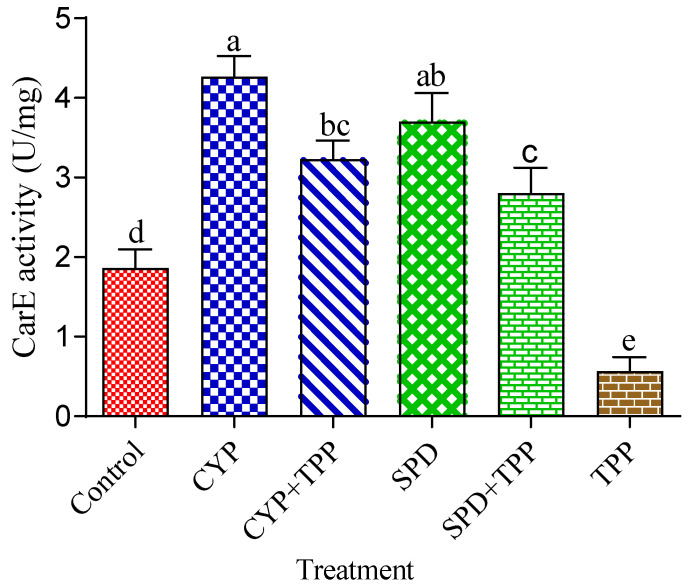
Effects of cypermethrin and spinosad (LC_50_) alone and together with triphenyl phosohate (TPP) on detoxification activities of CarE activity of the fourth instar of *S. littoralis* larvae. Each column represents the mean ± SEM of three independent experiments. Different letters on top of the bar indicate significant differences (*p* < 0.05). CYP: cypermethrin, SPD: spinosad.

**Figure 2:**
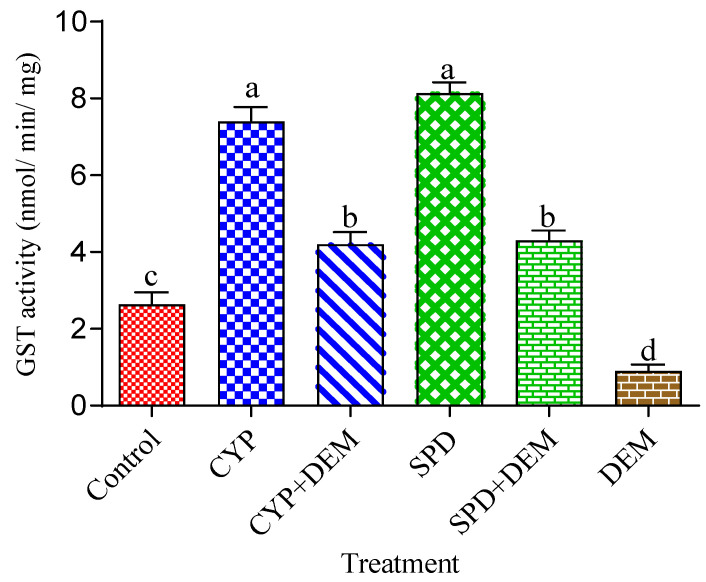
Effects of cypermethrin and spinosad (LC_50_) alone and together with diethyl maleate (DEM) on detoxification activities of Glutayhion S-transferase (GST) activity of the fourth instar of *S. littoralis* larvae. Each column represents the mean ± SEM of three independent experiments. Different letters on top of the bar indicate significant differences (*p* < 0.05). CYP: cypermethrin, SPD: spinosad.

**Figure 3:**
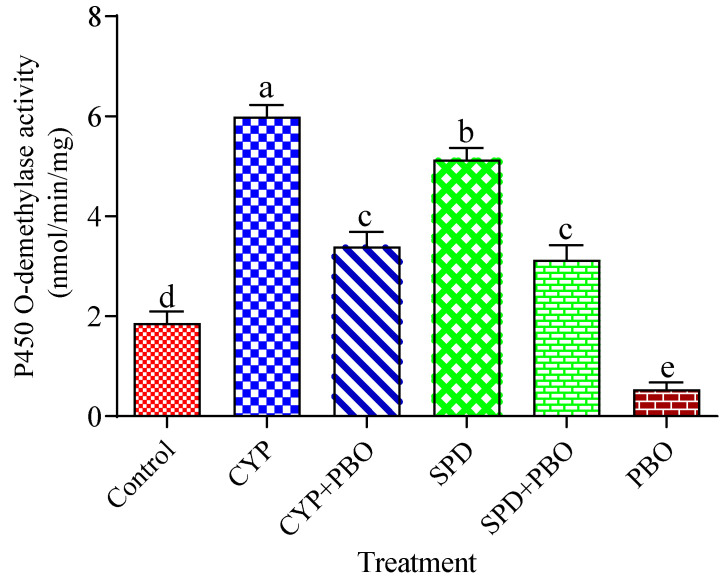
Effects of cypermethrin and spinosad (LC_50_) alone and together with piperonyl butoxide (PBO) on detoxification activities of cytochrome P-450 activity of the fourth instar of *S. littoralis* larvae. Each column represents the mean ± SEM of three independent experiments. Different letters on top of the bar indicate significant differences (*p* < 0.05). CYP: cypermethrin, SPD: spinosad.

The effects of enzyme inhibitors (TPP, DEM, and PBO) and the LC_50_ of tested insecticides (CYP and SPD) on the enzyme activities of CarE, GST, and Cyp 450 are illustrated in Figure 1, Figure 2 and Figure 3. CarE activities remarkably improved after treatment with CYP and SPD alone in comparison with the control. Moreover, CarE activities decreased with the addition of TPP plus the tested insecticides, as well as TPP alone (Figure 1). Meanwhile, Figure 2 indicates that the effect of DEM alone and in combination with CYP and CPD caused a significant (*p* < 0.05) reduction in GST level, but CYP and CPD exhibited a noticed elevation in GST level in comparison with control. Statistically significant differences were observed regarding the activity of Cyp 450 in all treatments (Figure 3). The treatment of tested insecticides alone improved the amount of Cyp 450 compared to the control. Meanwhile, PBO alone reduced the activity of Cyp 450 in comparison with PBO+ insecticides and the control.

## 4. Discussion

The substantial pesticide resistance resulting from the extensive use of synthetic insecticides must be addressed as soon as possible [27]. The results further demonstrated that the tested insecticides led to mortality on the fourth instar larvae of *S*. *littoralis*, where the LC_50_ of CYP and SPD decreased *S. littoralis* larvae. The mode of action of CYP is the disruption of voltage-gated sodium channel (VGSC) function [41]. Sodium channel function is disrupted by certain binding sites. The mechanisms of action for SPD are to disrupt the neural functions via an alteration of nicotinic receptor function [42,43]. This result validated the work of El-Sheikh [44], who reported that the LC_50_ value of CYP was 1.675 μg/mL against the newly molted fourth instar larvae of *S*. *littoralis*. Moreover, the LC_50_ value was 1.71 μg/mL one day after treatment with CYP for the Lab-SS of the fourth instar larvae of *S*. *littoralis* [1]. A similar result reported that the LC_50_ value of SPD was 0.50 μg/mL 48 h after treatment against the fourth instar larvae of *S*. *littoralis* [15]. Another study indicated that SPD had a toxic effect against *S*. *littoralis* with the LC_50_ value (2.11 mg a.i./kg diet) two days after treatment [45]. Additionally, the LC_50_ value of SPD against *S*. *littoralis* was 0.87 μg/mL 24 h after treatment [46].

The management of *S. littoralis* populations through the use of synthetic chemical pesticides could lead to the development of pesticide resistance [1]. Metabolic resistance generated by detoxifying enzymes is a key resistance mechanism. Consequently, identifying the primary detoxification enzyme responsible for insecticide metabolism will enable novel pest management options [31]. The biochemical mechanism of resistance demonstrated that PBO, TPP, and DEM significantly influenced the toxicity of CYP and SPD against the fourth larval instars of *S. littoralis* in comparison with the tested insecticides alone. This may be due to synergistic action when mixed with insecticides, which is usually attributed to the inhibition of a variety of detoxifying enzymes that are important for insects as they enable them to utilize these enzymes against insecticides [47]. A similar type of synergistic effect of PBO has been investigated in the pyrethroid resistance population of *Spodoptera litura*, fenvalerate resistance of *H. armigera*, and fipronil resistance of *Chilo suppressalis* and *Choristoneura rosaceana* [48,49]. Liu and Yue [50] demonstrated that PBO increased the SPD toxicity to houseflies of both permethrin-resistant and permethrin-susceptible strains. Wang et al. [51] documented that PBO had stronger synergism for SPD compared to TPP and DEM. The synergistic effect of DEM and TPP on CYP and fenvalerate insecticides has been reported in *Spodoptera litura* [48]. Furthermore, Khan et al. [52] revealed the high toxicity of CYP with PBO and DEM synergism in *Apis mellifera* and *Musca domestica*. The activities of CarE, GST, and Cyp 450 were inhibited when TPP, DEM, and PBO were added to the tested insecticides, respectively, in comparison with the tested insecticides alone. This is because enzyme inhibitors connect to detoxification enzymes and prohibit them from degrading pesticides [53].

Our result agrees with the previous results that PBO, DEM, and TPP had synergistic effects on CYP of 1.5-, 1.7-, and 2.3-fold against *Tuta absoluta* (Meyrick) and remarkably decreased the activity of CarE, GST, and Cyp 450 [54]. Moreover, the synergistic effects of PBO, DEM, and TPP plus CYP reduced the number of *Spodoptera litura* via the inhibition of CarE, GST, and Cyp 450 activity compared to CYP alone [55]. Another study reported that PBO, DEM, and TPP plus CYP reduced the activity of CarE, GST, and Cyp 450 in *Amsacta albistriga* [56]. Wang et al. [57] reported that the toxicity of SPD to the Xinjiang and Taian populations considerably increased following PBO treatments. PBO boosted SPD toxicity in the Taian population more than in the Xinjiang population, with synergistic ratios of 2.0 and 4.7 48 h after treatment against *Helicoverpa armigera*, respectively. Furthermore, the toxicity of SPD plus PBO and DEM increased in the susceptible and resistant strains of *Musca domestica* in comparison with SPD alone [58].

Few reports have referred to the synergistic effects of insecticide toxicity on the auxiliary fauna [59,60,61]. Wu and jiang [62] found that PPO, TPP, and DEM enhanced the susceptibility of *Pteromalus puparum* and *Diadromus collaris* to CYP. On the contrary, some studies reported that a synergist could be selected that was specifically targeted to the mechanism of resistance present in the pest species but not in the natural enemies. Therefore, the efficacy of an insecticide could be increased against the target pest and still keep natural enemy populations high [63,64,65].

Taken together, our research results lay the foundations for a deeper understanding of the mechanisms contributing to the adaptation of *S. littoralis* to different types of insecticides, which is of considerable significance regarding the development of effective pest management strategies. Briefly, the emergence of pesticide resistance in various insecticides results in onerous pest management activities in agriculture. Consequently, finding an appropriate synergist molecule to increase the toxicity of insecticides on insects is critical.

## 5. Conclusions

The emerging insecticide resistance among the insects for various insecticides or pesticides results in difficult challenges in pest management in the agriculture field. Hence, finding a suitable synergist compound that can enhance the toxicity of pesticides to insect pests is vital, but these compounds need to be as gentle as possible for the rest of the insects, especially to the pests’ natural enemies. The overall findings of the present study revealed that the combined action of TPP, DEM, and PBO with synthetic chemical insecticides (CYP and SPD) exhibited synergistic action versus the fourth larval instars of *S. littoralis* and inhibition of detoxification enzymes (CarE, GST, and Cyp 450). Furthermore, Cyp 450 played the most critical role in insect tolerance to the tested insecticides. Consequently, our findings provide useful information for resistance monitoring, as well as designing and developing an insecticide management strategy for controlling *S. littoralis*.

## Figures and Tables

**Table 1:** Stomach activity of cypermethrin and spinosad against the fourth instar of *Spodoptera littoralis* 24 h after treatment.

Compounds	Toxicity Regression Equation	LC_50_ (μg/mL)	95% Fiducial Limits (μg/mL)	χ2	r	df
Cypermethrin	y = 0.0506x + 3.086	2.861	2.460–2.883	0.90	0.94	4
Spinosad	y = 0.0551x + 2.7615	3.273	2.904–3.396	1.03	0.95	4

**Table 2:** Stomach activity of enzyme inhibitors (PBO, DEM, and TPP) against the fourth instar of *Spodoptera littoralis* 24 h after treatment.

Compounds	Toxicity Regression Equation	LC_50_ (μg/mL)	95% Fiducial Limits (μg/mL)	χ2	r	df
PBO	y = 0.0335x + 3.0098	236.2	228.7–239.2	4.74	0.98	4
DEM	y = 0.0585x + 0.4371	324.5	319.1–329.9	4.78	0.97	4
TPP	y = 0.0218x + 3.6632	245.8	241.9–249.3	5.06	0.98	4

PBO, piperonyl butoxide; DEM, diethyl maleate; TPP, triphenyl phosphate.

**Table 3:** Stomach activity of enzyme inhibitors (PBO, DEM, and TPP) plus the tested insecticides against the fourth instar of *Spodoptera littoralis* 24 h after treatment.

Compounds	Toxicity Regression Equation	LC_50_ (μg/mL)	95% Fiducial Limits (μg/mL)	χ2	r	df	Synergistic Ratio
Cypermethrin + PBO	y = 0.0561x + 3.4655	1.580	1.105–1.809	0.38	0.94	4	1.810
Cypermethrin + DEM	y = 0.0503x + 3.3235	2.267	2.176–2.311	0.70	0.98	4	1.262
Cypermethrin + TPP	y = 0.0555x + 3.3675	1.962	1.877–1.992	0.58	0.95	4	1.458
Spinosad + PBO	y = 0.051x + 3.2425	2.344	2.271–2.395	0.72	0.96	4	1.396
Spinsad + DEM	y = 0.0512x + 3.1425	2.653	2.580–2.717	0.84	0.95	4	1.233
Spinosad + TPP	y = 0.0542x + 3.0125	2.530	2.477–2.583	0.80	0.97	4	1.293

Synergistic ratio = LC_50_ of single compounds/LC_50_ of the mixture of compounds and synergists. PBO, piperonyl butoxide; DEM, diethyl maleate; TPP, triphenyl phosphate.

## Data Availability

All data and materials are included in the manuscript.
